# Unearthing Inequities in the Relationship between Multiple Sociodemographic Factors and Diverse Elements of Park Availability and Quality in a Major Southern Metropolitan Region

**DOI:** 10.3390/ijerph21020204

**Published:** 2024-02-09

**Authors:** Shirelle H. Hallum, Marilyn E. Wende, Farnaz Hesam Shariati, Kelsey M. Thomas, Anna L. Chupak, Eleanor Witherspoon, Andrew T. Kaczynski

**Affiliations:** 1 Department of Health Promotion, Education, and Behavior, Arnold School of Public Health, University of South Carolina, Columbia, SC 29208, USA; shallum@email.sc.edu (S.H.H.); farnaz@email.sc.edu (F.H.S.); kmt10@email.sc.edu (K.M.T.); alchupak@email.sc.edu (A.L.C.); ecw9@email.sc.edu (E.W.); 2 Department of Health Education and Behavior, College of Health and Human Performance, University of Florida, Gainesville, FL 32608, USA; marilyn.wende@ufl.edu

**Keywords:** environmental justice, equity, parks, physical activity, socioeconomic disparities

## Abstract

Parks are critical components of healthy communities. This study explored neighborhood socioeconomic and racial/ethnic inequalities in park access and quality in a large U.S. southeastern metropolitan region. A total of 241 block groups were examined, including 77 parks. For each block group, we obtained multiple sociodemographic indicators, including unemployment rate, education level, renter-occupied housing, poverty rate, and racial/ethnic minority composition. All parks were mapped using geographical information systems and audited via the Community Park Audit Tool to evaluate their features and quality. We analyzed seven diverse elements of park quality (transportation access, facility availability, facility quality, amenity availability, park aesthetics, park quality concerns, and neighborhood quality concerns), as well as an overall park quality score by calculating the mean for all parks within each block group. The mean percent of residents below 125% of the poverty level and the percentage of renter-occupied housing units were significantly higher among block groups with any parks in comparison to block groups with no parks. In addition, there were significant positive associations between park transportation access scores and both the percentage of residents with less than high school education and the percent identifying as non-Hispanic white. Moreover, there was a significant negative association between park amenity availability and the block group’s unemployed population. Further, a significant negative association between park aesthetics and the population with a lower than high school education percentage was observed. Revealed differences in park availability, park acreage, and park quality dimensions emphasized the need for targeted policy, programmatic, and infrastructure interventions to improve park access and quality and address health disparities.

## 1. Introduction

Parks are widely acknowledged as key components of healthy and thriving communities [1,2,3,4,5]. From a public health standpoint, parks can help facilitate the prevention and remediation of major health issues, such as stress, asthma, diabetes, cardiovascular disease, and cancer [6,7,8]. This is partly because parks are key destinations for walking and recreation [9,10] and provide several opportunities for physical activity (PA) among residents of all ages [11,12]. They are available within most communities at low or no cost, benefiting large segments of the population, especially disadvantaged groups [13]. Adding to this, engaging in regular outdoor exercise is a strong preventive and remedial strategy for mental health concerns (e.g., depression, stress) [14,15] and can likewise positively impact social outcomes (e.g., social cohesion, community connection) [16,17,18]. In summary, the presence of parks in a community offers numerous public health, environmental, and social benefits [19,20]. Since park use contributes to overall community well-being in the myriad ways discussed above, it is important to precisely measure diverse park attributes that can facilitate or deter use. Objective methods, including geographic data and observational audits, are becoming more accessible and adopted by researchers [21,22,23]. Using such methods, research suggests that the use of parks is a function of diverse park attributes, including park proximity, features, condition, safety, and aesthetics [24,25,26,27,28,29,30]. Indeed, the availability of nearby urban green space is important, but the composition and quality of local parks, including the facilities and amenities therein, may be equally influential on whether residents visit or use these valuable resources [31]. For example, according to McCormack et al. [32], factors such as safety, aesthetics, amenities, maintenance, and accessibility are crucial to encouraging park use, while unclean washrooms, litter, and vandalism can discourage people from using parks. Another study in two low-income neighborhoods found that park use was associated with greater nearby street walkability, fewer incivilities, and improved aesthetics [33].

A growing body of research has examined park access and quality inequalities based on factors such as neighborhood socioeconomic status (SES) and racial/ethnic composition [34,35,36,37,38,39,40]. Much of this research has focused on park availability [41,42,43,44,45,46,47,48]. For example, according to some studies, there is less availability of parks and recreational spaces in low-income and high-minority communities [41,43,45,49,50]. In contrast, other studies have found no differences in park availability among neighborhoods with varying incomes or racial compositions [34,51,52]. Some studies have also examined park facilities based on SES or the racial/ethnic composition of an area [39,53,54,55]. For instance, Cohen et al. [56] showed that the size and number of facilities in parks in neighborhoods with high-poverty levels were the same as those in low-poverty districts. Fewer studies about park equity have considered diverse elements of park quality, given that such data are more subjective and more challenging to collect [34,39,57,58]. For example, in a study of 144 parks in El Paso, Kamel et al. [35] reported that parks in the highest income census tracts had significantly fewer quality and safety concerns (e.g., litter, vandalism) in the park and surrounding neighborhood. Other studies have also emphasized the frequently observed variations in park quality by socioeconomic and race/ethnicity characteristics [53,59,60]. Observed inequities in the distribution of quality parks and their health-promoting facilities and amenities (e.g., playgrounds, restrooms, lighting) may contribute to rampant socioeconomic and racial/ethnic disparities in health behaviors and outcomes [61,62,63]. For example, as a result of reduced access to quality parks among racial and ethnic minority groups, past research documents that black residents used local parks occasionally or less often than white residents [55,64]. Such usage differences likely contribute to observed racial/ethnic disparities in the prevalence of meeting PA recommendations [65,66]. This effect is even stronger when considering income, with predominantly minority, low-income neighborhoods having reduced access to quality parks compared to higher-income areas [39,58]. Furthermore, as economic marginalization is prevalent in most metropolitan regions’ older inner cities and inner-ring suburbs, park acquisition and enhancement in these areas are often limited by local resources [67,68]. It is imperative to consider that these disparities significantly affect park use and health among different groups in the community.

While a growing body of evidence has examined inequities in park access, quality, and use, significant limitations exist. First, most studies examine cities, counties, or tracts, while few existing analyses take place at the census block group level, which more accurately represents a neighborhood and where the starkest disparities in park access and quality may be observed [58,69]. Second, existing research identifying disparities has predominantly focused on income and racial/ethnic disparities and fails to examine other important sociodemographic indicators (e.g., housing characteristics, unemployment, educational attainment) that may have differing relationships with the numerous subsets of park availability (e.g., total count of parks, park acreage) and quality (e.g., facilities, amenities, incivilities, aesthetics) [34,39,58,70]. Finally, most research has focused primarily on the availability or presence of park facilities, but it is important to also consider whether the quality of parks, including characteristics such as facilities, amenities, and issues in the surrounding neighborhood, is equitably distributed across communities [62,71]. 

Adding to the aforementioned literature gaps, very little research on racial/ethnic and socioeconomic disparities in park access and quality has specifically focused on the southeastern region of the United States (US) [34]. The southeastern region of the U.S. is known for high rates of chronic disease, greater racial/ethnic diversity, and pronounced income inequality [72]. Columbia, South Carolina, is located in the midlands of SC, a state where rates of physical inactivity (27.6% in SC vs. 25.3% nationally) and related chronic diseases are high [73,74]. Similar to statewide trends, disparities in chronic disease morbidity are also pronounced in Columbia (Eliminating Health Disparities [75]). The Columbia metropolitan region currently shows high rates of physical inactivity. This may be because there is low access to exercise opportunities, and 24.5% of residents do not have access to neighborhood parks or recreational facilities on average [76]. Columbia is the state capital and second-largest metropolitan area in the state, and South Carolina is one of the fastest-growing states in the country (with its population increasing by 10.7% from 2010 to 2020). Therefore, it is important to understand whether existing public infrastructure can support the growing and diverse population. 

Thus, the overall purpose of this study was to conduct an equity analysis regarding the presence and quality of health-promoting parks within residential areas in the Columbia metropolitan region of the southeastern region of the United States. Specifically, the study’s objectives were to (1) examine inequities in park availability (i.e., number of parks and park acreage) according to neighborhood unemployment, poverty, housing tenure, education, and racial and ethnic composition, and (2) examine inequities in multiple elements of park quality (i.e., transportation access, facility availability, facility quality, amenity availability, park aesthetics, park quality, and neighborhood quality) according to neighborhood unemployment, poverty, housing tenure, education, and racial and ethnic composition. 

## 2. Methods

### 2.1. Study Setting

**Figure 1:**
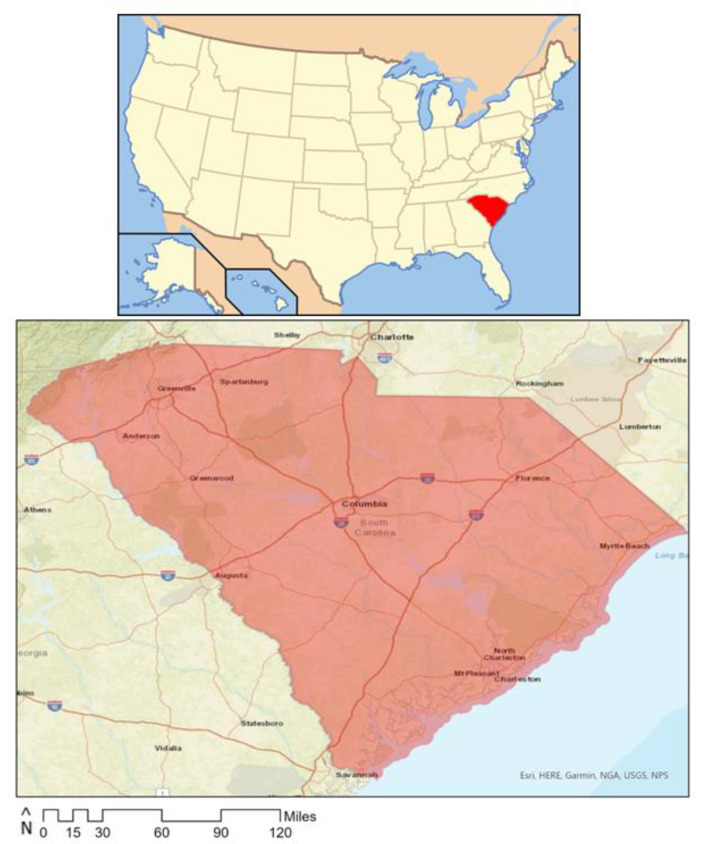
Study location, including Columbia, Cayce, West Columbia, and Forest Acres in South Carolina.

This study took place in 2021 in a large southeastern metropolitan region (Figure 1). The areas under study included Columbia, Cayce, West Columbia, and Forest Acres in South Carolina. According to rural–urban commuting area (RUCA) codes, which classify US census tracts with respect to their rural/urban status [77,78], almost all block groups were urban. These regions contained a 2019 estimated population of 315,639 people, a land area of 276.89 square miles, and 77 diverse neighborhood, regional, and specialty parks. The region had a diverse population, with 31.7% of residents identifying as black or African American and 57.3% as white, and citizens of all ages, including 24.8% aged 19 and under and 12.0% over the age of 65.

Neighborhoods were defined as census block groups for this study. Generally containing between 600 and 3000 people, census block groups are designed to provide granularity while maintaining anonymity [79]. The granularity of the data allows for a more comprehensive analysis of sociodemographic factors within smaller geographic areas, approximating neighborhood scales [79]. In total, 241 block groups were identified using ArcGIS that intersected the boundaries of the four study communities. The median household income of the included block groups ranged from $11,550 to $214,643, with more residents below the poverty line (19.7%) compared to the state average (13.8%) [80]. Overall, the study area exhibited significant sociodemographic diversity and provided an ideal setting to examine both income and racial/ethnic disparities in access to health-promoting resources.

### 2.2. Measures

#### 2.2.1. Neighborhood Sociodemographic Characteristics

**Figure 2:**
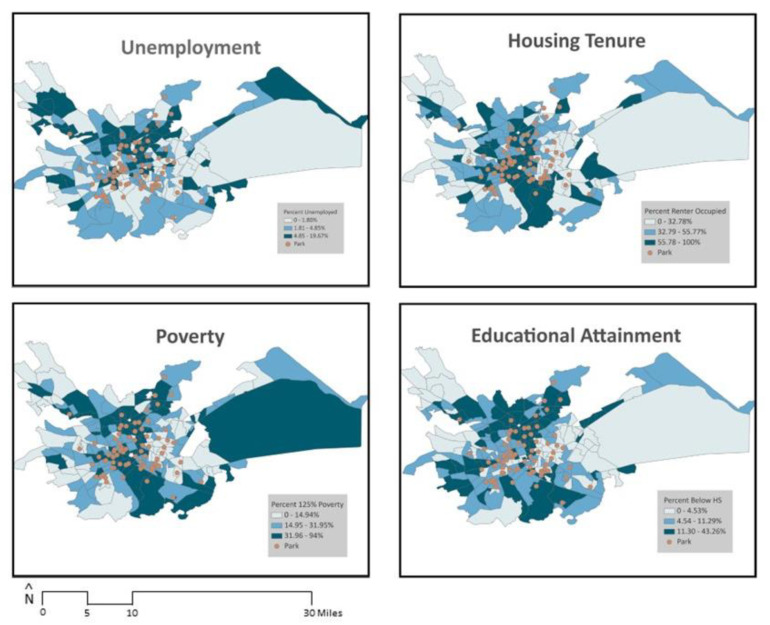
Socioeconomic characteristics and park locations (HS stands for High School).

**Figure 3:**
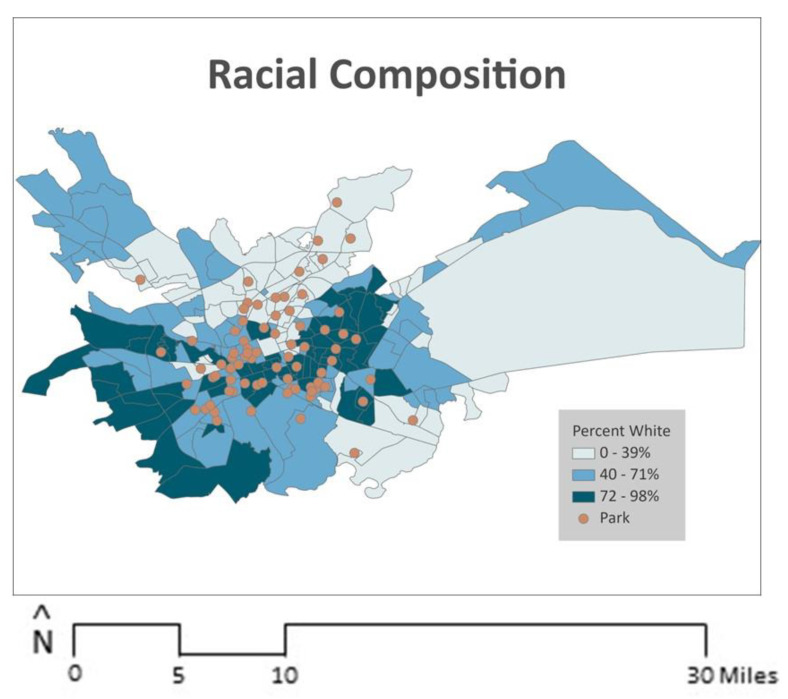
Racial composition and park location.

To examine indicators of socioeconomic status, consistent with prior research, four key economic and education variables were analyzed individually: (i) percent of the population that was unemployed; (ii) percent with less than high school education; (iii) percent of renter-occupied housing; and (iv) percent under 125% of the federal poverty threshold [34,81]. For each block group in the study area (*N* = 241), American Community Survey (ACS) 5-year estimates (2015–2019) were collected to measure each of the four variables. ACS data were also used to determine the block group-level racial/ethnic composition by calculating the number of non-Hispanic white residents divided by the total block group population. Figure 2 and Figure 3 illustrate the distribution of unemployment, poverty, renter-occupied housing, educational attainment, and race/ethnicity overlaid with park locations in the study area.

#### 2.2.2. Park Data Collection

A list of parks was gathered from the respective park departments of each city. This was cross-referenced using Google Maps to ensure that no parks in the study area were missing. A total of 77 parks, excluding community centers without greenspace, were identified. Objective audits of all 77 parks were performed using a Qualtrics-based online version of the Community Park Audit Tool (CPAT) [23]. CPAT facilitates the collection of park data in four categories: (i) park information (e.g., weather, map availability); (ii) access and surrounding neighborhood (e.g., transit stops, neighboring land uses); (iii) park activity areas (e.g., tennis court, green space); and (iv) park quality and safety (e.g., drinking fountain, shade, lighting) [23]. Approximately 10% of parks were assessed by two separate researchers to ensure inter-rater reliability, and all audited elements had at least 90% agreement between raters. 

To convert CPAT scores from the park level to the neighborhood (census block group) level, the aggregate points feature in GIS was used to determine which parks fell within each block group’s area (in rare cases of neighborhood overlap, each park was only allocated to the block group containing the largest section of the park’s area). Statistics about the parks in each block group were then calculated. First, as described below, each block group was assigned two park availability scores by summing the number of parks and the number of park acres therein. Second, an average score for each park quality indicator described below was calculated by taking the mean for all parks within the block group. 

#### 2.2.3. Park Availability

Park availability was assessed in two ways: as the sum of the number of parks per block group and as the number of acres of park space per block group. Specifically, each block group was then categorized as having at least one available park versus no parks and into low, intermediate, or high park acreage using tertiles. 

#### 2.2.4. Park Quality

As described below, CPAT data were used to calculate multiple metrics related to park quality as well as an overall score for each park based on the previously developed ParkIndex [82]. Each park quality metric comprised multiple indicators, which were weighted equally (due to the absence of specific evidence supporting the differential weighting of diverse elements of the parks).

***Transportation Access.*** Transportation access was measured through seven CPAT components: the presence or absence of adjacent sidewalks, bordering bike routes, traffic signals on adjacent roads, public transportation visible from the park, parking for vehicles, an external path connected to the park, and visible signs clarifying park attributes, such as park hours or rules. To arrive at the park-level transportation access score, the sum of these variables was divided by 7 and multiplied by 100 to reach a percentage (max = 100).

***Facility Availability.*** CPAT also captured the availability of 17 recreational facilities for PA within the park: playground, swing set, sport field, baseball field, swimming pool, splash pad, basketball court, tennis court, volleyball court, trail, fitness station, skate park, dog park, green space, lake, disc golf, and other designated PA areas, as specified by the individual auditing the park. A park was assigned a maximum facility score (100) if it contained at least seven of the different types of facilities; as such, the sum of these variables was divided by 7 and then multiplied by 100. 

***Facility Quality.*** Block groups with higher facility quality scores contained parks with more PA facilities that were both usable and in good condition (half a point for each criterion per facility). Usable facilities were defined as everything necessary for use being present and nothing preventing use, while those in good condition were defined as looking clean and maintained. For each park, a facility quality score was calculated by dividing the sum of all facility quality scores by the total amount of possible points (based on the number of facilities present) and multiplying by 100 (max = 100). 

***Amenity Availability.*** CPAT also assessed the availability of amenities that would support visitation, including restrooms, drinking fountains, lights, picnic tables, benches, and trash cans within the park. To arrive at the park-level amenity availability score, the sum of these variables was divided by 6 and multiplied by 100 (max = 100). 

***Park Aesthetics.*** Parks were also evaluated for the presence of seven aesthetically pleasing features, such as evidence of landscaping, artistic features, historical/education features, wooded areas, trees throughout, water features, and meadows. A park was allotted a maximum score if it had at least five of these features. To arrive at the park aesthetics score, the sum of these variables was divided by 5 and multiplied by 100 to reach a percentage (max = 100). 

***Park Quality Concerns.*** The presence of eight quality concerns was captured for each park: graffiti, vandalism, litter, animal waste, excessive noise, poor maintenance, evidence of threatening persons/behavior, or dangerous spots in the park. To arrive at the total park quality score, the eight variables were summed, divided by 8, multiplied by 100, and then subtracted from 100 (to be recoded in a similar direction to the other park quality variables described here; max = 100). 

***Neighborhood Quality Concerns.*** Likewise, we evaluated the presence of several quality concerns in the neighborhood immediately surrounding and visible from the park: poor lighting, graffiti, vandalism, litter, heavy traffic, noise, vacant or unfavorable buildings, poorly maintained properties, lack of eyes on the street, or evidence of threatening persons or behavior. To arrive at the total neighborhood quality concerns score, the 10 variables were summed, divided by 10, multiplied by 100, and then subtracted from 100 (to be recoded in a similar direction to the other park quality variables described here; max = 100).

***Total Park Quality Score.*** Finally, to arrive at a total park quality score for each park (out of 100), the mean of scores for transportation access, facility availability, facility quality, amenity availability, park aesthetics, park quality concerns, and neighborhood quality concerns were calculated [82].

## 3. Analysis

A one-way analysis of variance (ANOVA) was used to examine differences in sociodemographic indicators (percent of residents unemployed, percent of the population below the poverty line, percent of housing units that are renter-occupied, percent with less than high school education, and percent non-Hispanic white) according to park availability (none vs. any parks). In addition, a one-way ANOVA was used to examine differences in the same sociodemographic indicators according to park acreage (low, intermediate, or high) after excluding block groups with no parks. If assumptions were violated (e.g., distributions have the same variance), Welch’s variance-weighted one-way ANOVA was used. 

A linear regression was used to assess the relationships between the block group sociodemographic indicators (i.e., percent unemployed, percent with less than high school education, percent of housing units that are renter-occupied, percent of the population below the poverty line, and percent non-Hispanic white) and the total park quality score and individual components of park quality (scores out of 100 for transportation access, facility availability, facility quality, amenity availability, park aesthetics, park quality concerns, and neighborhood quality concerns). All analyses controlled block group population density, and tests were considered significant at *p* < 0.05. Geographic representations were produced using ArcGIS Pro (Environmental Systems Research Institute, Redlands, CA, USA).

## 4. Results

### 4.1. Park Availability

Table 1 presents differences in park availability by block group sociodemographic characteristics. A total of 241 residential block groups were included, distributed across the four cities. The average number of parks in each block group was 0.41, with a range from 0 to 4. 

Only the mean percentage of residents below 125% of the poverty level differed by park availability (F = 4.2, *p* = 0.04). Specifically, there was a greater percentage of residents below 125% of the poverty level in block groups with any parks (M = 30.9%, SD = 19.8) compared to block groups with no parks (M = 25.1%, SD = 20.2).

Table 2 presents differences in block group sociodemographic characteristics according to tertiles (low, intermediate, and high) of park acreage among only block groups containing parks. A total of 73 residential block groups were included. There were no significant differences in sociodemographic variables according to park acreage categories (Table 2). 

### 4.2. Park Quality Indicators

Table 3 presents associations between the seven elements of park quality as well as the overall park quality score and sociodemographic characteristics. 

#### 4.2.1. Park Transportation Access 

There were significant positive associations between the park transportation access scores and both the percentage of residents with less than high school education (B = 0.45, *p* = 0.02) and the percent identifying as non-Hispanic white (B = 0.34, *p* = 0.05). 

#### 4.2.2. Facility Availability 

There were no significant relationships between block group characteristics and facility availability. 

#### 4.2.3. Facility Quality

There were no significant associations between block group characteristics and facility quality (Table 3). 

#### 4.2.4. Park Amenity Availability

There was a significant negative association between park amenity availability and the block group’s unemployed population (B = −0.26, *p* = 0.03). 

#### 4.2.5. Park Aesthetics

There was a significant negative association between park aesthetics and the percentage of the population with less than high school education (B = −0.56, *p* ≤ 0.01).

#### 4.2.6. Park Quality Concerns

There were no statistically significant associations between park quality concerns and block group characteristics. 

#### 4.2.7. Neighborhood Quality Concerns

There were no significant relationships between block group characteristics and neighborhood quality concerns. 

#### 4.2.8. Overall Park Quality

**Figure 4:**
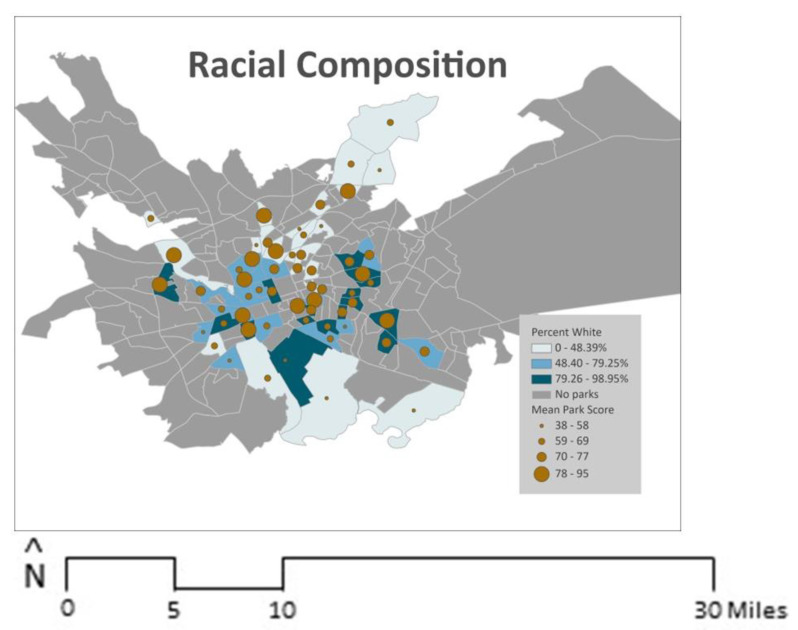
Racial composition and park quality.

**Figure 5:**
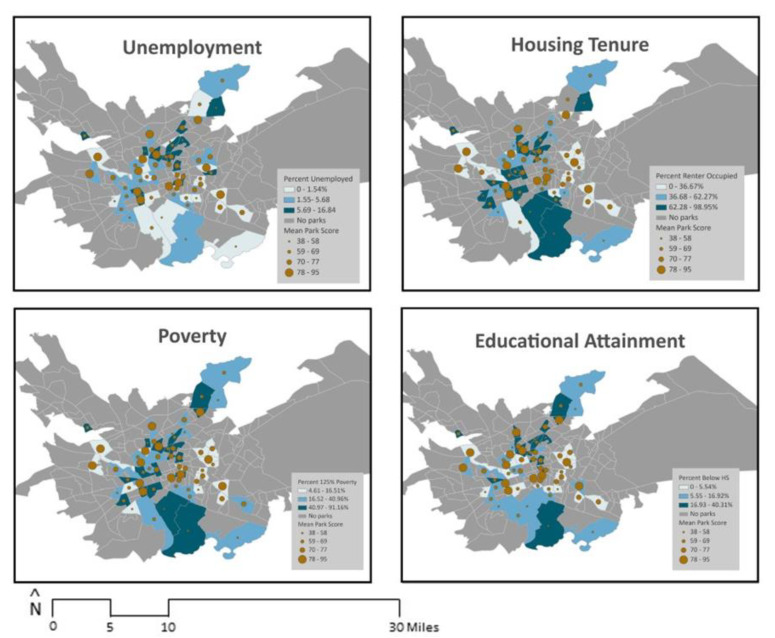
Socioeconomic characteristics and park quality (HS stands for High School).

Finally, Figure 4 and Figure 5 depict block group characteristics and mean overall park quality scores. There were no significant associations between block group characteristics and overall park quality (Table 3). 

## 5. Discussion

Parks are essential components of a healthy community, as they are valuable resources for PA and other aspects of physical and mental health [12,83,84]. Using a large southeastern metropolitan area as a case study, this study provided an equity analysis of the availability and quality of health-promoting parks across residential zones. We examined not only park availability and quantity of acreage but also a wide array of park quality indicators and how all of these park elements were associated with numerous socioeconomic characteristics of the neighborhood, including unemployment, education, housing tenure, poverty, and race/ethnicity. Developing interventions and policies that promote PA, reduce chronic diseases, and improve overall well-being among populations experiencing health disparities requires a comprehensive understanding of key neighborhood resources [85]. Policymakers, urban planners, and public health advocates can work collaboratively to design more inclusive and equitable parks that facilitate active lifestyles and enhance community well-being by identifying park features and attributes that are lacking in underserved regions [86,87]. Research like this contributes to efforts to create healthier communities, specifically in the southeastern region of the U.S., where obesity and related chronic diseases are prevalent [88].

According to our results about park availability, higher levels of poverty and renter-occupied housing were correlated with greater access to parks. Previous studies have been mixed, with many reporting no relationship between elements of neighborhood disadvantage and the number of parks [34,51] or that more disadvantaged areas had greater access to parks [45,54,89]. We also found no significant differences in sociodemographic variables according to park acreage. If general access to parks explained the relationship between socioeconomic disadvantage and lower PA engagement, an inverse relationship would be expected; however, indicators of lower SES were associated with greater access to parks in the current study. It is possible that parks are not distributed according to present-day socioeconomic patterns. For example, many urban core areas in the U.S., where population characteristics are often diverse, underwent development when integrated planning and mixed land-use strategies were prevalent. This development pattern often included parks and green spaces, along with residential, commercial, and industrial land uses [90,91], thus leaving a legacy of green space in central areas that are often more diverse and disadvantaged. 

Using detailed observational audits, we also investigated diverse aspects of park quality. For transportation access, we found that a greater percentage of residents with lower educational attainment and more white residents were associated with increased transportation access to parks. These results are consistent with findings from other studies. For example, Kelly et al. [92] reported that in the St. Louis metropolitan area, African American block groups were fifteen times more likely to have street segments with lots of obstructions for safe walking. Similarly, low-SES neighborhoods and a higher proportion of black residents were associated with poor sidewalk quality, according to recent research by Rajaee et al. [93].

Furthermore, we found a significant negative association between park amenity availability and the block group’s percentage of unemployed population, as well as a similar negative association between park aesthetics and the percentage of residents with less than high school education. Some past research has reported similar findings about high-SES neighborhoods having greater aesthetics and amenities (e.g., trees and ponds) than low-SES areas [34,54,70]. However, other studies found no socioeconomic disparities in park aesthetics or amenities [50,94]. In areas with better park aesthetics, residents and local organizations may collaborate to improve park features through active community involvement; in contrast, community engagement and resources to advocate for park improvements may be lacking in neighborhoods with lower educational attainment [95,96,97], resulting in inequities and a dearth of supporting amenities and aesthetically pleasing parks. It is worth mentioning that the reasons why some of our results differed from some past findings could be due to differences in study design and methodologies, sample characteristics, and time frame. For instance, Rigolon et. al. [98] showed that depending on how researchers measure the quality of parks, findings could vary between inequity versus equity or mixed. 

Regarding other findings, there were no statistically significant variations in facility quality, park quality, neighborhood quality concerns, or overall park quality scores by block group characteristics. One study conducted by Engelberg et al. [58] in the Seattle, WA, and Baltimore, MD, regions concluded that lower-income neighborhoods had better park quality on average. These disparate findings might be due to the different settings in which the studies were conducted. Regardless, the quality of park environments, including facilities and amenities, is important because much research suggests residents visit and use park areas based on their quality [26,99,100,101]. It is possible that shifts in funding mechanisms may have aggravated park inequalities, but local examination of such issues is warranted [98,102,103].

## 6. Implications for Research and Practice

This study demonstrated the value of examining the varying relationships between sociodemographic factors and park availability and quality in a major southern metropolitan region. Objective tools and metrics, like GIS and CPAT, used in this research can facilitate the identification and remediation of park access disparities within and across communities. They can also provide a transparent way to visualize inequalities that allows researchers, citizens, and other key stakeholders to work together toward redressing these concerns [82]. In another perspective, improving park and recreation accessibility is one strategy for better serving disadvantaged residents [104], which may explain positive correlations between higher levels of unemployment and greater transportation access. The more white residents, the higher the transportation access, as has been seen with some other neighborhood amenities [92], which also outlines a need for policy interventions to ensure equitable park accessibility. Such issues are important because various aspects of transportation access (e.g., street connectivity, sidewalks, traffic speed) can impact both the frequency of park visits and the use of parks for PA [105]. Initiatives such as Safe Routes to Parks that help improve transportation access can improve equity and safety and facilitate greater park use and PA [106]. In addition, researchers, public health specialists, parks and recreation professionals, and urban designers can replicate the methods used in this research to inform interventions and equity planning tools [107].

Regarding implications for research, the methodology used in this study could be replicated in other locations. The study results highlight the need for more nuanced research on sociodemographic factors, park availability, and park quality. Research should be conducted to determine how access to park facilities may differ across segments of the population, given that parks are used differently by various gender, age, and racial/ethnic groups [12]. In addition, exploring contextual factors impacting park availability could be another avenue for researchers to consider. Such factors could include resource allocation, historical contributors, community engagement, and other policies and practices that can facilitate more equitable and inclusive approaches to park planning and development [108,109]. Another important research implication could concentrate on the importance of exploring various initiatives in park equity to promote health (e.g., equitable park funding, community engagement strategies, targeted accessibility enhancements), as the provision of green space and parks can help address disparities in physical and mental health [110].

With respect to practice implications, these results can provide valuable evidence and inform efforts to reduce disparities in access to health-promoting resources in the southeastern region of the U.S. Such evidence may be critical in order to stir community action, activate political sentiment, and justify policy and practice interventions related to improving park access and quality. For example, identifying disparities related to transportation access to parks may point to the need for targeted investments in public transportation infrastructure so that all residents can easily access recreational places. For instance, in one study by Schultz et al. [111], the installation of a signalized pedestrian crosswalk over a five-lane major highway increased safe access to parks. Likewise, identified disparities in facility quality may spur investments in renovations. These strategies could involve revitalizing abandoned spaces or turning vacant lots into community green spaces [112]. For example, a study conducted by Veitch et al. [113] showed that the installation of a well-designed playscape increased park visits and promoted PA. Similarly, a study by Cohen et al. [114] found that playgrounds with an increased variety of play elements had more visitors. In other scenarios, improvements to the health-promoting potential of parks may be identified, such as implementing community garden spaces, outdoor group exercise classes, art installations, and walking trails [115,116,117]. For any such interventions to be culturally relevant and address specific community needs, it is critical to engage local communities throughout the park planning and development process [118,119]. Practitioners can create park designs and programs that reflect residents’ diverse preferences and interests in conjunction with community organizations and stakeholders [120]. In addition, city authorities in the major southern metropolitan region could use such findings to enhance strategies regarding park quality and availability. Identifying underserved neighborhoods with low availability or quality of parks can help in prioritizing resources for renovating parks, enhancing their conditions, or creating new local green spaces.

## 7. Strengths and Limitations 

This study had several strengths. First, it focused on a specific context, including Columbia, Cayce, West Columbia, and Forest Acres in South Carolina, which are largely urban. The diversity of this region’s sociodemographic, socioeconomic, and health disparity characteristics allows for greater insights into park availability and quality inequities and their potential impact on health behaviors and outcomes. Another strength was the use of rigorous measurement methods, such as GIS and CPAT, to assess inequities in park availability and multiple elements of park quality. Finally, this study is intended to generate a framework for future research that connects multiple interdisciplinary realms, such as urban planning, public health, sociology, and environmental science, enabling a comprehensive examination of the interplay between sociodemographic factors, park availability, and quality. The combination of various disciplines lends robustness to the findings, rendering them amenable to practical application by a broad spectrum of stakeholders, including policymakers, urban planners, public health professionals, park and recreation managers, and community advocates. 

Despite its strengths, this study had some limitations. First, although the methods employed herein can be used to assess park access and quality disparities in other locations across the U.S., the results likely cannot be generalized to all places and may be region-specific. Second, subjective assessments, such as gathering the opinions or perceptions of park users or residents about park availability and quality, would add additional insights into park awareness and equity. Third, factors such as park programming, staffing, and supervised activities were not taken into account, though some research suggests that these elements may be important for promoting park-based PA [118]. Fourth, our study adopted an equal weighting approach in considering multiple indicators for each aspect of park quality. This aligns with standard practices in composite measures of park quality, where equal weighting is often chosen to maintain equity in the assessment process. However, we recognize the potential for variation in the importance of different indicators across diverse contexts, and future research may wish to explore alternative weighting schemes tailored to each park element and setting. Finally, in this study, we controlled block group population density but did not categorize block groups, which all fell within city boundaries according to urbanicity (almost all were classified as urban according to RUCA codes). However, future research in more diverse settings should consider classifying block groups based on RUCA (or other) codes and proceed with analyses according to urban/rural status. Moreover, incorporating outlying rural areas would help in better understanding how to address the most underserved and marginalized communities. That said, it is less feasible to use environmental audits to conduct such large-scale evaluations, so finding ways to scale up these assessments is crucial.

## 8. Conclusions

This study examined socioeconomic and racial/ethnic disparities in park access and quality, specifically focusing on a large metropolitan region in South Carolina. We conducted this study to explore the distribution and quality of health-promoting parks in residential areas of a region where rates of PA are disproportionately low and health disparities are high. We examined an array of factors, including unemployment, education, housing tenure, poverty, and race/ethnicity, associated with park disparities. Using rigorous methods and detailed census block group-level data, the findings revealed numerous disparities in park availability and quality, highlighting the need for targeted interventions to promote equitable access to health-promoting parks and improve community well-being in the Columbia metropolitan area. Such data enable decision-makers to make informed choices and prioritize resource allocation to increase the accessibility and quality of parks at the population level, thus providing residents, regardless of socioeconomic or racial/ethnic background, with healthier, happier, and more vibrant communities. Overall, similar methods should be replicated in other urban areas across southern metropolitan regions to address persistent health, PA, and park access disparities.

## Figures and Tables

**Table 1:** Differences in characteristics of study area block groups by park availability (*N* = 241).

Block Group Characteristics	Overall (*n* = 241)		
	Block Groups with No Parks	Block Groups with Any Parks	F (*p*) ^1^
**Total *N* (%)**	168 (69.7)	73 (30.3)	
**Percent of Residents Unemployed** ***Mean (SD)***	4.0 (3.9)	4.1 (3.8)	0.1 (0.79)
**Percent of Residents Below 125% of the Poverty Level** ***Mean (SD)***	**25.1** **(20.2)**	**30.9** **(19.8)**	**4.2** **(0.04)**
**Percent of Housing Units Rented** ***Mean (SD)***	46.1 (26.4)	51.3 (25.7)	2.0 (0.16)
**Percent of Residents with Less Than High School Education** ***Mean (SD)***	9.7 (9.3)	11.2 (10.4)	1.3 (0.26)
**Percent of Residents Identifying as Non-Hispanic White**	53.1 (31.4)	53.9 (34.3)	0.3 (0.86)

Bolded values represent significance with α = 0.05; ^1^ one-way analysis of variance (ANOVA) estimates

**Table 2:** Differences in characteristics of study area block groups by park acreage (*N* = 241).

Block Group Characteristics	Overall (*n* = 241)			
	Low	Int.	High	F (*p*) ^1^
**Total *N* (%)**	23 (31.5)	25 (34.3)	25 (34.3)	
**Percent of Residents Unemployed *Mean (SD)***	4.8 (5.2)	3.6 (2.7)	4.2 (3.3)	0.6 (0.57) ^2^
**Percent of Residents Below 125% of the Poverty Level** ***Mean (SD)***	27.5 (21.3)	28.2 (17.1)	36.8 (20.4)	1.7 (0.19)
**Percent of Housing Units Rented** ***Mean (SD)***	47.8 (25.3)	48.6 (23.1)	57.3 (28.5)	1.0 (0.37)
**Percent of Residents with Less Than High School Education** ***Mean (SD)***	10.3(9.8)	11.7 (11.3)	11.5 (10.3)	0.1 (0.88)
**Percent of Residents Identifying as Non-Hispanic White *Mean* (SD)**	51.4 (37.6)	54.5 (34.8)	55.6 (32.0)	0.1 (0.91)

Int. = intermediate; bolded values represent significance with α = 0.05; ^1^ one-way analysis of variance (ANOVA) estimates; ^2^ Welch’s ANOVA used to account for violation of homogeneity of variance assumption.

**Table 3:** Differences in characteristics of study area block groups by park quality indicators (*N* = 241).

	Overall (*n* = 241)							
Block Group Characteristics	Transportation Access B (*p*)	Facility Availability B (*p*)	Facility Quality B (*p*)	Amenity Availability B (*p*)	Aesthetics B (*p*)	Park Quality Concerns B (*p*)	Neighborhood Quality Concerns B (*p*)	Total Park Quality Score B (*p*)
**Percent of Residents Unemployed**	0.13 (0.32)	−0.13 (0.31)	0.11 (0.37)	**−0.26** **(0.03)**	−0.08 (0.51)	0.10 (0.42)	0.25 (0.04)	−0.03 (0.80)
**Percent of Residents Below 125% of the Poverty Level**	−0.10 (0.60)	0.02 (0.94)	0.22 (0.27)	−0.22 (0.22)	0.18 (0.31)	−0.09 (0.61)	−0.29 (0.12)	−0.03 (0.87)
**Percent of Housing Units Rented**	−0.06 (0.73)	−0.22 (0.24)	−0.22 (0.24)	−0.15 (0.37)	0.09 (0.59)	−0.16 (0.345)	−0.12 (0.49)	−0.20 (0.28)
**Percent of Residents with Less Than High School Education**	**0.45** **(0.02)**	0.35 (0.06)	−0.19 (0.30)	0.19 (0.26)	**−0.56** **(<0.01)**	−0.20 (0.24)	−0.18 (0.30)	−0.03 (0.87)
**Percent of Residents Identifying as Non-Hispanic White**	**0.34** **(0.05)**	−0.07 (0.70)	0.12 (0.50)	0.14 (0.40)	−0.02 (0.90)	0.14 (0.39)	−0.10 (0.56)	0.13 (0.44)

Bolded values represent significance with α = 0.05.

## Data Availability

Data used in this study are available from the authors upon request.
